# Cold at the Core: Osborn Waves in Neurosarcoidosis-Induced Central Hypothermia

**DOI:** 10.1155/2019/5845839

**Published:** 2019-01-15

**Authors:** Gregory Scott Troutman, Jason Salamon, Matthew Scharf, Jeremy A. Mazurek

**Affiliations:** ^1^Sidney Kimmel Medical College at Thomas Jefferson University, Philadelphia, PA, USA; ^2^Department of Medicine, Division of Cardiology, Morristown Medical Center, Atlantic Medical Group, Morristown, NJ, USA; ^3^Department of Medicine, Division of Sleep Medicine, Robert Wood Johnson University Hospital, New Brunswick, NJ, USA; ^4^Department of Medicine, Division of Advanced Heart Failure and Transplant Cardiology, Hospital of the University of Pennsylvania, Philadelphia, PA, USA

## Abstract

Osborn waves, or J waves, initially described by John Osborn in 1953 in hypothermic dog experiments, are highly sensitive and specific for hypothermia. Initially thought to be secondary to a hypothermia-induced “injury current,” they have more recently been attributed to a voltage differential between epicardial and endocardial potassium (I_to_) currents. While the exact conditions required to induce such waves have been debated, numerous clinical scenarios of environmental and iatrogenic hypothermia have been described. Below, we report a novel case of hypothermia—that of neurosarcoidosis-induced central hypothermia with resultant Osborn waves and other associated findings found on electrocardiogram (ECG).

## 1. Introduction

Osborn waves, or J waves, initially attributed to a hypothermia-induced “injury current,” have more recently been attributed to a differential between epicardial and endocardial potassium (I_to_) currents creating a voltage gradient and the observed J wave [1, 2]. While not pathognomonic for hypothermia, the presence of J waves, most commonly seen in the anterior and lateral precordial leads, is highly sensitive and specific for hypothermia [3, 4].

Neurosarcoidosis is a relatively uncommon, often debilitating condition affecting approximately 5% of patients with sarcoidosis. Pituitary disease can cause thyroid, gonadal, and adrenal abnormalities or panhypopituitarism. Hypothalamic involvement can further result in central diabetes insipidus and dysthermia (either hypothermia or hyperthermia). Management often involves systemic corticosteroids and the replacement of all deficient hormones to correct the endocrine dysfunction [5].

Below, we describe the case of a patient with neurosarcoidosis who presented with hypothermia and prominent Osborn waves on electrocardiogram (ECG) in the setting of significant pituitary and hypothalamic dysfunction.

## 2. Case Report

**Figure 1:**
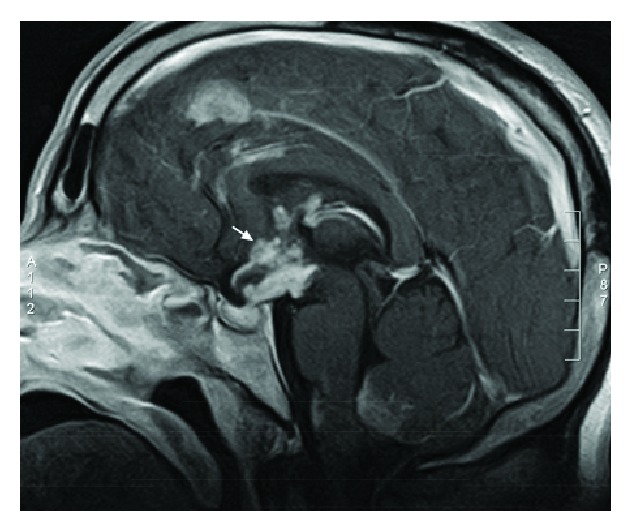
Brain magnetic resonance image showing neurosarcoidosis involving the hypothalamus and pituitary.

**Figure 2:**
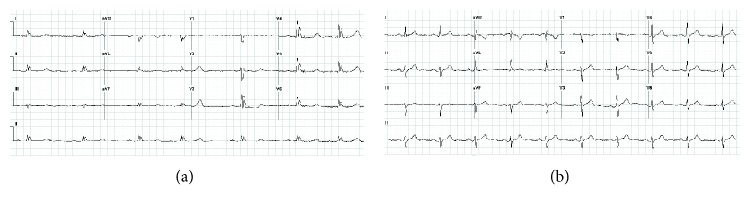
(a) Electrocardiogram at 32°C revealing sinus bradycardia with premature atrial contractions, Osborn waves most prominent in the precordial lateral leads, prolonged PR and QT intervals. (b) Electrocardiogram after initiation of treatment reveals an increase in heart rate to 60 beats per minute and an improvement of the PR (210 ms), QRS (98 ms), and QT (QTc 432 ms) intervals.

A 40-year-old African-American male with neurosarcoidosis involving the hypothalamus and pituitary (Figure 1) presented to an urban academic medical center with altered mental status. On arrival, the patient was lethargic but responsive to verbal stimuli and the physical examination was otherwise unremarkable. The patient was noted to be hypothermic (32°C) and hypernatremic (176 mEq/L). Admission ECG revealed sinus bradycardia at 41 beats per minute, first-degree AV block (PR interval 280 ms), premature atrial contractions, prolonged QRS (160 ms) and QT (QTc 584 ms) intervals, and Osborn waves most prominent in the precordial lateral leads (Figure 2(a)).

The patient was admitted to the intensive care unit where careful intravenous fluid management and administration of intranasal desmopressin were initiated. After 24 hours, with the improvement of his serum sodium, the patient's mental status improved. The patient was warmed via external warming blankets, with resolution of the above electrocardiographic findings (Figure 2(b)). The remainder of the patient's treatment included corticosteroids, testosterone, levothyroxine, and desmopressin, and he was discharged home ten days after presentation.

## 3. Discussion

Neurosarcoidosis is a debilitating condition that affects a minority of patients (~5%) with sarcoidosis. As a systemic granulomatous disease, sarcoidosis can affect both the central and peripheral nervous systems with the neurological manifestations varying depending on the areas of disease involvement [6, 7]. Pituitary disease and hypothalamic involvement can result in severe endocrine dysfunction including panhypopituitarism and central diabetes insipidus and dysthermia (either hypothermia or hyperthermia), respectively, as were seen in this patient [4–7].

Osborn waves, or J waves, initially described as a hypothermia-induced “injury current,” have more recently been attributed to a differential between epicardial and endocardial potassium (I_to_) currents creating a voltage gradient and the observed J wave [1, 2]. Though not necessarily pathognomonic for hypothermia, the presence of J waves is highly sensitive and specific for hypothermia. Most commonly seen in the anterior and lateral precordial leads is the amplitude of the J wave which is inversely proportional to the degree of hypothermia and is present in 80% of individuals with a temperature below 35°C. Other electrocardiographic findings include PR, QRS, and QT prolongation, as in this patient, as well as supraventricular and ventricular arrhythmias in more severe hypothermia [3].

Since its original description, it has been suggested that hypothermia must be accompanied by some other disturbance (i.e., acidosis) to result in the development of the Osborn wave [1, 3]. Subsequent reports, however, have suggested a hypothalamic or neurogenic cause for the J waves as seen in patients with subarachnoid hemorrhage [3, 8]. Experimental animal experiments inducing subarachnoid hemorrhage have confirmed similar ECG findings to that of hypothermia suggesting a possible relationship between hypothalamic dysfunction and autonomic catecholamine imbalance with a resultant disorder of myocardial rhythm and function [9, 10].

Thus, our patient represents an unusual cause of hypothermia with classic findings on ECG. These findings, unlike prior reports, may represent effects of both systemic hypothermia and hypothalamic dysfunction. To our knowledge, this is the first report of neurosarcoidosis-induced hypothermia with associated Osborn waves along with other associated ECG changes, all of which resolved with warming, steroid and hormone replacement, and supportive care.
